# Do Changes in Oral Microbiota Correlate With Plasma Nitrite Response? A Systematic Review

**DOI:** 10.3389/fphys.2019.01029

**Published:** 2019-08-13

**Authors:** Khrystyna Zhurakivska, Giuseppe Troiano, Vito Carlo Alberto Caponio, Mario Dioguardi, Luigi Laino, Angela Bruna Maffione, Lorenzo Lo Muzio

**Affiliations:** ^1^Department of Clinical and Experimental Medicine, University of Foggia, Foggia, Italy; ^2^Multidisciplinary Department of Medical-Surgical and Odontostomatological Specialties, University of Campania “Luigi Vanvitelli”, Naples, Italy

**Keywords:** oral microbiota, oral bacteria, nitrite (nitrate) [NO_2_^−^ (NO_3_^−^)], mouthwash, nitric oxide—NO

## Abstract

**Background:** Nitric Oxide (NO) has a role in immunitary defense, regulation of mucosal blood flow and mucus production, regulation of smooth muscle contraction, cerebral blood flow, glucose regulation, and mitochondrial function. NO can be synthetized endogenously through the L-arginine-NO pathway or it can be absorbed by the human intestine through the dietary intake. Most of the ingested NO is in the form of nitrate (${\text{NO}}_{3}^{-}$). ${\text{NO}}_{3}^{-}$ is a substrate of oral and intestinal microbiota and, at the end of the catabolic pathway, NO is released. Using antibacterial mouthwashes leads to an alteration of salivary ${\text{NO}}_{3}^{-}$ metabolism, however, with unclear consequences on the circulating NO levels. The aim of this study is to perform a systematic review in order to elucidate if the alterations of oral microbiota lead to modifications in plasma NO content.

**Methods:** Electronic databases were screened, using the following terms: [“oral bacteria” and (nitrate OR nitrite OR nitric)]. Clinical studies reporting ${\text{NO}}_{3}^{-}$ and ${\text{NO}}_{2}^{-}$ measurements in blood and their correlation to oral microbiota variations were included. We focused on the correlation between the changes in oral microbiota and plasma concentrations of nitrites (primary outcome). Subsequently, we investigated if modifications in oral microbiota could lead to changes in blood pressure and salivary ${\text{NO}}_{2}^{-}$ concentration (secondary outcome).

**Results:** Six studies, for a total of 82 participants were included in this review. In four studies, the use of mouthwash correlated to a reduction of plasma nitrite concentration (*p* < 0.05); Two studies did not find any difference in plasma nitrate or nitrite concentration. In five studies, a correlation between blood pressure (BP) changes and antibacterial mouthwashing emerged. Anyway, only three studies suggested a significant increase of systolic BP following mouthwashing compared with controls.

**Conclusions:** Although, the role of oral bacteria has been unequivocally demonstrated in the regulation of salivary ${\text{NO}}_{3}^{-}$ metabolism, their influence on plasma concentration of NO species remains ambiguous. Further studies with larger sample size are required in order to demonstrate if an alteration in oral microbiota composition may influence the blood content of ${\text{NO}}_{3}^{-}$/${\text{NO}}_{2}^{-}$/NO and all the linked biological processes.

## Introduction

The specific contributions of oral microbiota in human physiopathology has not been explored yet, although different studies report how the composition of oral microbiota has a role in oral and systemic diseases (Santarelli et al., 2015; Sampaio-Maia et al., 2016; Aarabi et al., 2018; Cardoso et al., 2018).

In particular, some resident bacteria in the oral cavity are able to reduce the dietary intake of nitrates (${\text{NO}}_{3}^{-}$), producing nitrites (${\text{NO}}_{2}^{-}$). Among these, *Neisseria, Veillonella, Haemophilus, Porphyromonas, Fusobacterium, Prevotella, Leptotrichia, Brevibacillus, and Granulicatella* are mainly involved in this process (Doel et al., 2005; Hyde et al., 2014). The main dietary source of nitrate is represented by vegetables (Ysart et al., 1999). Once ingested, nitrate is absorbed in the gastrointestinal tract and enters in the bloodstream. Here, it mixes to the endogenous nitrate, which mainly derives from the L-arginine-NO pathway (Leaf et al., 1989). Most of the nitrate is then excreted in the urine, while up to 25% of plasma nitrate is taken up by the salivary glands and placed in the enterosalivary circulation (Spiegelhalder et al., 1976). Salivary nitrate is metabolized to nitrite by oral commensal bacteria and it can be further reduced to nitric oxide (NO). ${\text{NO}}_{2}^{-}$ can be stored in blood and tissues and used when there is a decreased endogenous production of NO (Bryan and Loscalzo, 2011). Consequently, NO may contribute to a myriad of biological processes, among which: immunitary defense, regulation of mucosal blood flow and mucus production, regulation of smooth muscle contraction, cerebral blood flow, glucose regulation, and mitochondrial function (Bryan and Loscalzo, 2011). The above described processes are known as “nitrate-nitrite-nitric oxide pathway” (Zweier et al., 1995). Considering this physiological pathway, the oral microbiota gained a fundamental role; changes in its composition were assumed to affect the final NO production (Hyde et al., 2014). In particular, since the 1980s, it has been proposed that antimicrobial mouthwashing could inhibit the nitrite formation in saliva (Tannenbaum et al., 1976). Subsequently, it was supposed that such alterations could also lead to variations in the systemic availability of NO (Govoni et al., 2008; Kapil et al., 2013; Woessner et al., 2016). Plasma analysis may contribute to better understand the NO blood availability because of changes in the oral microbiota.

The aim of this study is to perform a systematic review in order to elucidate if alterations of oral microbiota lead to modifications in plasma content of nitrogen species.

## Materials and Methods

The protocol for this systematic review followed the PRISMA (Preferred Reporting Items for Systematic Reviews and Meta-Analyses) guidelines (Liberati et al., 2009).

In addition, it was prospectively registered on the online database PROSPERO (International prospective register of systematic reviews) with the registration number CRD42019124473.

This systematic review was performed in order to answer the following question: “Do changes in oral microbiota influence variations in blood content of nitrogen species?”

Electronic databases PubMed, SCOPUS, and Web of Science were screened independently by two authors, KZ and GT, in order to select studies suitable for inclusion in this review. The following strategy of search was used: [“oral bacteria” AND (nitrate OR nitrite OR nitric)]. In addition, bibliographies of systematic reviews and included studies were manually revised in order to find other articles to be included in this study.

Only studies published in English language and fulfilling the following criteria were considered eligible for inclusion in this review:

- Original clinical studies reporting ${\text{NO}}_{3}^{-}$ and ${\text{NO}}_{2}^{-}$ blood measurements correlated with oral microbiota variations.

No restrictions were applied about the year of publication. Studies eligibility was independently assessed in a joint session by two authors (KZ and GT). Authors screened for articles by reading only title and abstract of the studies, according to the eligibility criteria. If the data reported in abstract were not sufficient to make a clear decision, the full text publication was examined. The selected papers were full-text evaluated in a second round and, if fulfilling the inclusion criteria, were included in the qualitative analysis. Any disagreement was solved in a discussion between reviewers in a joint session.

The following information was extracted from the included papers:

- Authors' names and year of publication, study design, number of participants, type of intervention, plasma ${\text{NO}}_{3}^{-}$ and ${\text{NO}}_{2}^{-}$ changes, salivary ${\text{NO}}_{3}^{-}$ and ${\text{NO}}_{2}^{-}$ changes, Blood Pressure Changes, Relative abundance of ${\text{NO}}_{3}^{-}$ reducing species.

We focused on the correlation between the changes in oral microbiota and plasma concentrations of nitrites (primary outcome). Subsequently, we also investigated if modifications in oral microbiota could lead to changes in blood pressure and salivary ${\text{NO}}_{2}^{-}$ concentration (secondary outcome).

## Results

**Figure 1:**
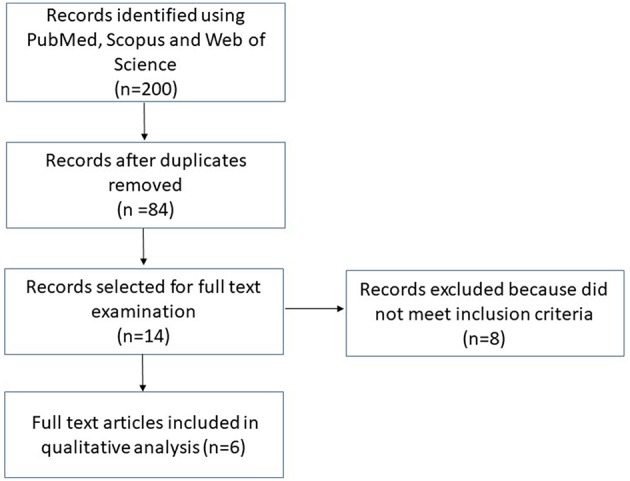
Flow-chart summarizing the selection process.

A total of 200 records were retrieved after the application of search strategy. After the first screening and duplicates removing, 84 records were chosen for title and abstract evaluation. Fourteen studies, resulting after this step, were selected for full text examination. Eight studies were excluded because did not meet inclusion criteria (Lundberg et al., 2004; Doel et al., 2005; Hyde et al., 2014; Clodfelter et al., 2015; Qu et al., 2016; Koch et al., 2017; Kapil et al., 2018; Preshaw, 2018). The reasons for exclusion are detailed in Table 1. The flow-chart in Figure 1 summarizes the selection process of inclusion. At the end, six papers were included in the systematic review (Table 2; Govoni et al., 2008; Kapil et al., 2013; Bondonno et al., 2015; McDonagh et al., 2015; Sundqvist et al., 2016; Woessner et al., 2016). In these studies, the test groups (mouthwashing) were compared to control (no mouthwashing) and implications on the production of nitrogen species were evaluated. Other three studies with a different design were identified and deemed valid to be included in the present review (Burleigh et al., 2018; Vanhatalo et al., 2018; Liddle et al., 2019). They only investigated how changes in oral microbiota could lead to alterations in ${\text{NO}}_{3}^{-}$/${\text{NO}}_{2}^{-}$ concentrations in human fluids. They were considered relevant for the development of the topic and are showed separately in the results (Table 3).

**Table 1:** Summary of excluded studies.

**Number**	**References**	**Reasons for exclusion**
1	Clodfelter et al., 2015	Do not deal with oral microbiota changes.
2	Kapil et al., 2018	Do not deal with oral microbiota changes.
3	Doel et al., 2005	Do not report the blood content of NOx species.
4	Hyde et al., 2014	Do not report the blood content of NOx species.
5	Lundberg et al., 2004	Not original study.
6	Qu et al., 2016	Not original study.
7	Koch et al., 2017	Not original study.
8	Preshaw, 2018	Not original study.

**Table 2:** Summary of data extracted from the included studies.

**References**	**N^**°**^ of participants**	**Study design**	**Type of intervention**		**Salivary ${\text{NO}}_{2}^{-}$ changes**	**Salivary ${\text{NO}}_{2}^{-}$ changes**	**Plasma ${\text{NO}}_{2}^{-}$ changes**	**Plasma ${\text{NO}}_{2}^{-}$ changes**	**Blood pressure changes**	**Relative abundance of ${\text{NO}}_{2}^{-}$ reducing species**
			**Test (T)**	**Control (C)**						
Govoni et al., 2008	7	Cross-over	MW (10 ml of Corsodyl) before ingestion of sodium nitrate (10 mg/kg in 100 ml water)	Ingestion of sodium nitrate (10 mg/kg in 100 ml water)	No significant difference between groups	Abolished immediately after MW and remained below basal levels during the entire observation period	No significant difference between groups	The rise in test group was markedly attenuated	Not determined	The bacterial counts were reduced by a mean of 80% after the mouth wash
Kapil et al., 2013	19	Cross-over	MW (10 ml Corsodyl 0.2% chlorhexidine) twice daily for 7 days	No intervention	No changes in Control group; Increase in Test group (***p*** **<** **0.001**)	No changes in Control group; Decrease in Test group (−282 ± 35 μmol/L, ***p*** **<** **0.001)**	No changes in Control group; Increase in Test group (***p*** **=** **0.0048**)	No changes in Control group; Decrease in Test group (−71 ± 15 nmol/L, ***p*** **=** **0.001**)	Increasing of clinic SBP in Test group (***p*** **=** **0.003**)	Not determined
McDonagh et al., 2015	12	Crossover	STRONG MW (Corsodyl®); WEAK; (Vademecum med® non-chlorexidine) 3 times daily, followed by BR consuming (70 mL containing ~6.2 mmol of NO3^−^) twice daily	Deionised water, followed by BR consuming (70 mL containing ~6.2 mmol of NO3^−^) twice daily	At 2 h: the increase was greater in STRONG compared to CON (***P*** **<** **0.05**) but not WEAK (*P* > 0.05). At 4 h the increase was significantly larger in STRONG compared to CON and WEAK **(*****p*** **<** **0.05**)	The increase was significantly lower at 2 h in STRONG compared to CON (*P*<**0.05**). At 4 h, was lower after STRONG compared to CON and WEAK (*p*<**0.05**)	The increase was not significantly different between CON, WEAK, and STRONG at 2 or 4 h post final BR ingestion	At 2 h, the elevation was lower in STRONG compared to CON (by 200 ± 174 nM) and WEAK (by 89 ± 112 nM) (***P*** **<** **0.05**); change was lower (by 110 ± 157 nM) in WEAK compared to CON at 2 h (***P*** **<** **0.05**). At 4 h was higher in CON and WEAK, compared to STRONG (***P*** **<** **0.01**).	No differences in resting BP changes (*P* > 0.05). During treadmill walking, the increase in SBP and MBP was higher 4 h after the final nitrate bolus in STRONG compared with CON (***P*** **<** **0.05**) but not WEAK	Not determined
Bondonno et al., 2015	15	Cross-over	Antibacterial MW 1.28 mg/mol chlorhexidine Gluconate twice daily for 3 days	Tap water	Increase (MW: 686 μmol/L; control: 252 μmol/L, ***p*** **<** **0.001**)	Decrease (mouthwash: 41 μmol/L; control: 111 μmol/L, ***p*** **=** **0.01**)	No difference (MW: 34.6 μmol/L, control: 30.0 μmol/L, *p* = 0.2)	A trend toward decreased not reaching statistical significance (MW: 150 nmol/L, control: 180 nmol/L, *p* = 0.09)	Test group compared to control: increase in SBP (2.3 mm Hg; ***p*** **=** **0.01**) but did not increase DBP (0.7 mm Hg; *p* = 0.4)	Not determined
Sundqvist et al., 2016	17	Crossover	MW (2 g chlorhexidine, 0.55 g menthol, 70 ml 95% ethanol and 930 ml distilled water per 1,000 ml total volume) three times a day for 3 days	MW without clorexidine three times a day for 3 days	Higher in Test: 1,118 μM; placebo: 401 μM, ***p*** **<** **0.01**	Lower in Test: 23 μM; placebo: 248 μM, ***p*** **<** **0.001**	No difference (Test: 23 μM; placebo: 22 μM)	No difference: (Test 0.21 μM; placebo 0.22 μM)	No significant changes	Not determined
Woessner et al., 2016	12	Crossover	Two 70 mL bottles of BR juice for a total of 8.4 mmol nitrate intake, followed by 1) Listerine® antiseptic mouthwash (active ingredients: Eucalyptol 0.092%, Menthol 0.042%, Methyl salicylate 0.060%, Thymol 0.064%), 2) Cepacol ® antibacterial mouthwash (active ingredients: Cetylpyridinium chloride 0.05%), 3) Chlorhexidine mouthwash (active ingredient: chlorhexidine gluconate 0.12%)	Two 70 mL bottles of BR juice for a total of 8.4 mmol nitrate intake, followed by water	No differences among treatments (*p* = 0.33)	1) Antiseptic MW lower than control treatment **(*****p*** **<** **0.05**); 2) Antibacterial MW lower than antiseptic (***p*** **<** **0.05**); 3) Chlorexidine MW lower than antiseptic (***p***** <0.05**), but not significantly lower than Antibacterial (p = 0.07)	No effect for MW treatments (*p* = 79)	1) antiseptic MW not significantly different than control (*p* = 0.45); 2) Antibacterial MW lower than control (***p*** **<** **0.05**); 3) chlorhexidine MW lower than control (***p*** **<** **0.01**)	SBP significantly higher in Antibacterial and Chlorexidine conditions than in control **(*****p*** **<** **0.01**) and Antiseptic (***p*** **<** **0.05**) conditions	Not determined

*MW, mouthwash; BP, Blood Pressure; SBP, Systolic Blood Pressure; DBP, diastolic blood pressure; MBP, mean Blood Pressure. Statistically significant values are in bold*.

**Table 3:** Summary of data extracted from studies evaluating oral microbiota composition.

**References**	**N^**°**^ of participants**	**Study design**	**Type of intervention**		**Relative abundance of ${\text{NO}}_{2}^{-}$ reducing species**	**Salivary ${\text{NO}}_{2}^{-}$ changes**	**Salivary ${\text{NO}}_{2}^{-}$ changes**	**Plasma ${\text{NO}}_{2}^{-}$ changes**	**Plasma ${\text{NO}}_{2}^{-}$ changes**
			**Test**	**Control**					
Vanhatalo et al., 2018	18	Crossover	Two 10-day dietary supplementation periods with NO3^−^ (NO3^−^ -rich concentrated beetroot juice (BR) (2 × 70 ml d−1, each 70 ml containing ~6.2 mmol ${\text{NO}}_{3}^{-}$	Two 10-day supplementation periods with NO3^−^ -depleted concentrated beetroot juice placebo (PL) (2 × 70 ml d−1, each 70 ml containing ~0.01 mmol ${\text{NO}}_{3}^{-}$)	${\text{NO}}_{3}^{-}$ supplementation altered the salivary microbiome compared to placebo by increasing the relative abundance of Proteobacteria (+225%) and decreasing the relative abundance of Bacteroidetes (−46%; *P* < 0.05). After NO^3−^supplementation the relative abundances of Rothia (+127%) and *Neisseria* (+351%) were greater, and *Prevotella* (−60%) and *Veillonella* (−65%) were lower than in the placebo condition (all ***P*** **<** **0.05**)	Not determined	Not determined	A significant correlation between plasma[${\text{NO}}_{2}^{-}$]/${\text{NO}}_{3}^{-}$ dose and %relative abundance of taxonomic units: - *Campylobacter concisus*: −0.55 (***p*** **<** **0.05**) - *Prevotella* (genus) −0.49 (***p*** **<** **0.05**) - *Prevotella melaninogenica* −0.57 **(*****p*** **<** **0.05**); - *Fusobacterium nucleatum* subsp. Vincentii: 0.55 (***p*** **<** **0.05**)	
Burleigh et al., 2018	25		Ingestion of 2 × 70 ml of NO3^−^ –rich beetroot juice (~12.4 mmol NO3^−^)		*Prevotella melaninogenica* 31.43 ± 10.33; *Veillonella dispar* 19.30± 11.97; *Hemophilus parainfluenzae* 2.78 ± 3.83; *Neisseria subflava* 2.57 ± 5.5.2; *Veillonella parvula* 0.24 ± 0.46; *Rothia mucilaginosa* 0.37 ± 0.49; *Rothia dentocarinosa* 0.003 ± 0.004	Not correlated with % relative bacteria abundance (*r* = 0.30; *p* = 0.17)	Correlated with % relative bacteria abundance (*r* = 0.44; *p* = 0.03)	Not correlated with % relative bacteria abundance (*r* = −0.25; *p* = 0.25)	Not correlated % relative bacteria abundance (*r* = −0.06; *p* = 0.78)
Liddle et al., 2019	10	Crossover	Ingestion of 2 × 70 ml of BR (total of ~12.4 mmol ${\text{NO}}_{3}^{-}$		*Prevotella melaninogenica* 23.8 ± 6.4; *Veillonella dispar* 13.0 ± 4.0; *Haemophilus parainfluenzae* 6.5 ± 5.9; *Neisseria subflava* 1.7 ± 1.0; *Veillonella parvula* 0.9 ± 0.4; *Rothia mucilaginosa* 0.2 ± 0.1; *Rothia dentocariosa* < 0.01 ± <0.01	Not determined	Not correlated with the sum of the NO3^−^ –reducing bacteria (*P* > 0.2) Abundance of *Neisseria subflava* was negatively associated with peak salivary [NO2^−^] (*R* = −0.43, *P* = 0.03)	Not determined	Not correlated with the sum of the ${\text{NO}}_{3}^{-}$ reducing bacteria Abundance of *Neisseria subflava* was negatively associated with plasma [NO2^−^] (*R* = −0.43, *P* = 0.03)

[NO2^−^]/NO3^−^ dose = plasma [nitrite] relative to nitrate dose per kg body mass ingested; *p <0.05. Statistically significant values are in bold.

The designs of the experiments were heterogeneous among studies, so it was not possible to perform a quantitative analysis. A total of 82 participants were enrolled in the included studies. The effect of various mouthwashes on ${\text{NO}}_{3}^{-}$ metabolism was investigated, providing discordant results among the studies. Four studies (Govoni et al., 2008; Kapil et al., 2013; McDonagh et al., 2015; Woessner et al., 2016) detected a significant negative influence of mouthwashing on plasma nitrite concentration (*p* < 0.05), meanwhile other two studies (Bondonno et al., 2015; Sundqvist et al., 2016) found no significant alteration of plasma nitrate and nitrite, comparing mouthwash administration against placebo.

The types of mouthwashes used in the studies were different; nevertheless all of them contained chlorhexidine. In two studies more than one mouthwash was tested (McDonagh et al., 2015; Woessner et al., 2016), resulting in some differences in the outcomes (Table 2). Results regarding the secondary outcome:

- Effects of mouthwashing on the salivary ${\text{NO}}_{2}^{-}$ changes: there were significant differences between the test and the control groups in all the studies (Govoni et al., 2008; Kapil et al., 2013; Bondonno et al., 2015; McDonagh et al., 2015; Sundqvist et al., 2016; Woessner et al., 2016).
- Effects of mouthwashing on Blood Pressure were evaluated in five studies: in three studies, Systolic Blood Pressure resulted significantly higher in the test groups compared to the controls (Kapil et al., 2013; Bondonno et al., 2015; Woessner et al., 2016). One study did not report any difference between test and control group (Sundqvist et al., 2016). In one study, the differences emerged only in some conditions (i.e., during treadmill walking), but not in others (McDonagh et al., 2015).

Three of the included studies, identified the types of oral nitrate reducing species and correlated their abundance with plasma ${\text{NO}}_{2}^{-}$ changes (Burleigh et al., 2018; Vanhatalo et al., 2018; Liddle et al., 2019). The most abundant species emerged to be: *Prevotella melaninogenica, Veillonella dispar, Hemophilus parainfluenzae, Neisseria subflava, Veillonella parvula, Fusobacterium nucleatum* subsp. nucleatum, *Campylobacter concisus, Leptorichia buccalis, Prevotella* intermedia. In two of these studies no correlation emerged between the abundance of ${\text{NO}}_{3}^{-}$ reducing species and plasma ${\text{NO}}_{2}^{-}$ changes (Burleigh et al., 2018; Liddle et al., 2019). Furthermore, in one study emerged how ${\text{NO}}_{3}^{-}$ supplementation influenced the salivary microbiome, leading to an increase in the relative abundance of *Proteobacteria* (+225%) and decrease of *Bacteroidetes* (−46%; *P* < 0.05; Vanhatalo et al., 2018). High relative quantities of *Rothia* and *Neisseria* and low presence of *Prevotella* and *Veillonella* were correlated with greater increases in plasma [${\text{NO}}_{2}^{-}$] in response to ${\text{NO}}_{3}^{-}$ supplementation (Vanhatalo et al., 2018).

## Discussion

Numerous bacteria express nitrate reductase genes and are capable to reduce nitrate to nitrite *in vitro* (Torres et al., 2016). In humans a physiologically relevant nitrate reduction occurs by means of some facultative anaerobic bacteria localized in crypts of the tongue (Duncan et al., 1995; Doel et al., 2005). Among the highest nitrate-reducers species, identified in the oral cavity, there are: *Firmicutes* (*Staphylococcus, Streptococcus*, and *Veillonella*) and *Actinobacteria* (*Actinomyces*), followed by numerous other taxa, including *Pasteurella, Rothia, Neisseria, Haemophilus, Granulicatella* (Smith et al., 1999; Palmerini et al., 2003; Hyde et al., 2014). Using dietary nitrate supplementation as selective pressure for bacteria capable of nitrate reduction, several studies found proliferation of *Veillonella* (Koopman et al., 2016), *Neisseria* (Velmurugan et al., 2016), and *Rothia* species (Velmurugan et al., 2016), all previously identified as high nitrate reducers. These data, as well as those of the studies examined in this review, confirm a close link between the oral microbiota and the salivary circulation of nitrates. However, the scientific evidence of a systemic repercussion is still poor today.

In order to evaluate the efficacy and/or alteration of conversion of oral nitrate to estimate the availability of NO pool, most of the studies, including our analysis, used plasma nitrite concentration (or changes in concentration), since the direct measurement of NO is difficult. This approach is justified by the relationships between plasma nitrite concentrations and physiological effects, as demonstrated by James et al. (2015). For this reason, only studies reporting blood measurements of nitrogen species were included in this review. Given this, the aim of the review was to verify even the alteration of oral microbiota could have a direct impact on plasma changes in nitrite concentration and, as proposed by some studies (Bondonno et al., 2015; Tribble et al., 2019), repercussions on Blood pressure or other biological processes (Joshipura et al., 2017).

From our analysis emerged some discordant results. Most of the evaluated studies report a correlation between oral microbiota and nitrites variations in plasma (Govoni et al., 2008; Kapil et al., 2013; McDonagh et al., 2015; Woessner et al., 2016). Two studies found no effect of mouthwashing on changes in plasma nitrites (Bondonno et al., 2015; Sundqvist et al., 2016). Govoni et al. (2008), despite having found a marked attenuation after mouthwash, noted that a significant rise in plasma nitrite occurred. Interestingly, this occurred despite the fact that the nitrite formation in the mouth has been completely abolished by the intervention, excluding its salivary origin. They proposed the existence of an alternative origin of nitrite, giving two possible explanations for this phenomenon: in the first hypothesis, they supposed a possible contribute of the gastrointestinal microflora to the nitrate reduction; in the second one, they hypothesized that mammalian cells in the gut wall o in another district are capable of nitrate reduction. However, despite this interesting hypothesis, results show that the sudden rise in plasma nitrite after nitrate ingestion is mainly deriving from bacterial nitrate reduction in the oral cavity and the use of antibacterial mouthwashes may have an impact on this chain. Even if, results of the interventions analyzed in the present review are statistically significant, the clinical repercussions need further investigation. Among these, the effects of the mouthwashes on Blood Pressure were those more analyzed, given the strong effect that the intake of rich in nitrates food has on blood pressure (Ashworth and Bescos, 2017). Following mouthwashing, a certain increase in blood pressure occurred in three studies (Kapil et al., 2013; Bondonno et al., 2015; Woessner et al., 2016), although, it should be noted that in one of these studies (Bondonno et al., 2015), the increase in BP was not associated with a statistically significant reduction in plasma nitrites. This controversial result could be linked to a bias in the kind of the patients included. The experiments were conducted in hypertensive men and women already in treatment with antihypertensive medication.

Another important risk of bias in interpreting the results, is given by the heterogeneity of the experiments conducted in various studies, in particular by the different timing of the fluid analysis, which could lead to mismatch in the comparison of the results, since the nitrate metabolism is subject to sudden changes in association with time.

## Conclusions

Although, most of the included studies demonstrate a statistically significant correlation between an alteration of the oral microbiota due to the use of antibacterial mouthwash and plasma changes in nitrite concentration, this link has not been established yet. Further RCT studies with larger samples and well defined designs are necessary in order to demonstrate if an alteration in oral microbiota composition may influence the blood content of ${\text{NO}}_{3}^{-}$/${\text{NO}}_{2}^{-}$/NO and, consequently, all the biological processes that depends on them.

## Data Availability

All datasets for this study are included in the manuscript and the supplementary files.

## Author Contributions

KZ and GT performed online research and collected data. VC analyzed data and results. LLa and MD contribute to write the manuscript. LLo and AM conceived and supervised the research.

### Conflict of Interest Statement

The authors declare that the research was conducted in the absence of any commercial or financial relationships that could be construed as a potential conflict of interest.
